# Supplementary material for: The interactome of intact mitochondria by cross-linking mass spectrometry provides evidence for coexisting respiratory supercomplexes
Source: Mol Cell Proteomics. 2017 Dec 8;17(2):216–32. doi: 10.1074/mcp.RA117.000470 (PMC5795388; doi:10.1074/mcp.RA117.000470)
Supplement: Supplemental Data [file supp_RA117.000470_133922_0_supp_23978_fzffwf.zip › spectra_annotation/mito_DR_spectra_annotation/421-1-4-1-4-1.pdf]

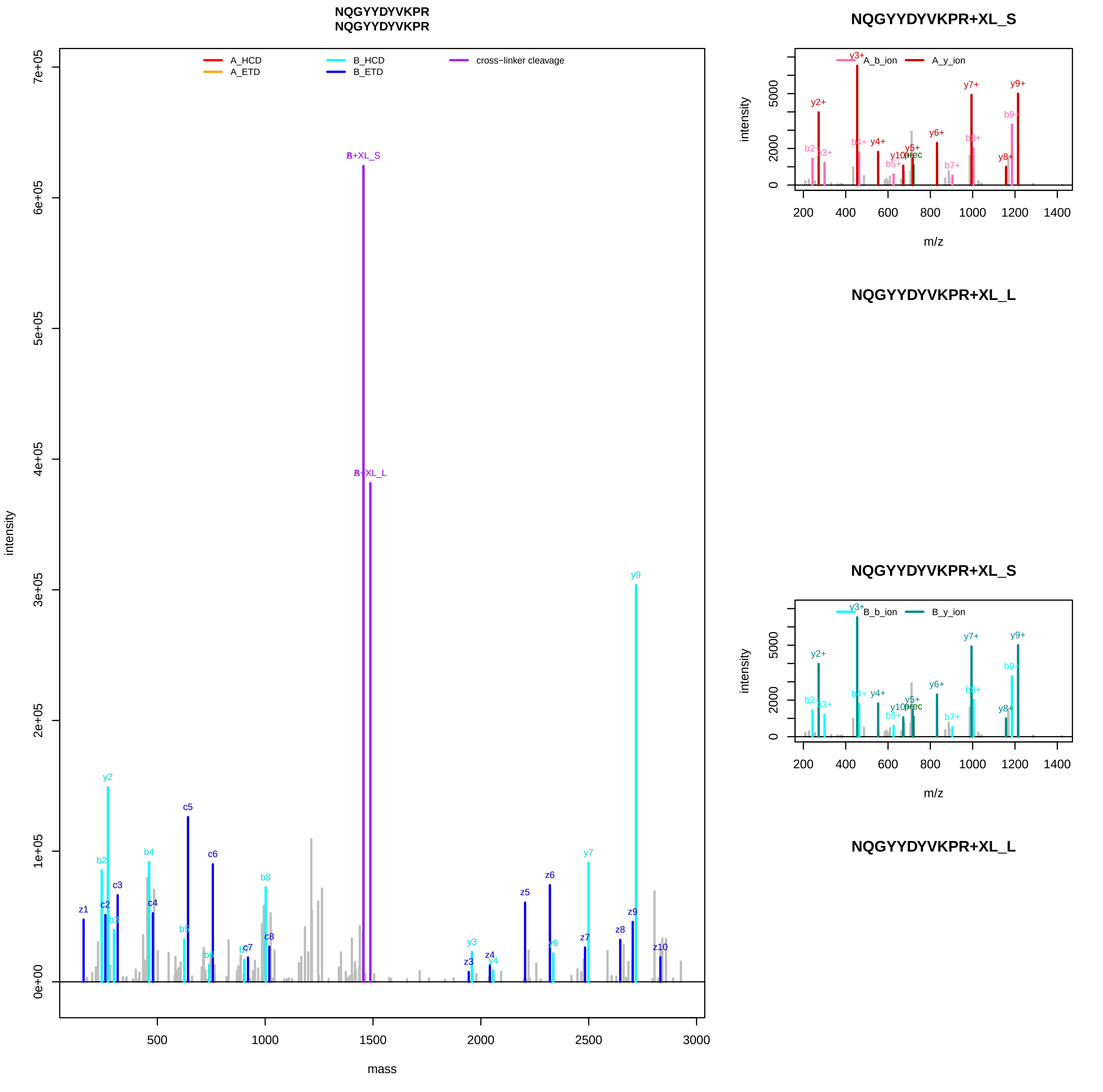
NQGYYDVKPR
NQGYYDVKPR
A_HCD
A_ETD
B_HCD
B_ETD
cross−linker cleavage
intensity
mass
B+XL_S
B+XL_L
z1 c2 b2 y2 b3 c3 b4 c4 b5 c5 b6 c6 b7 c7 b8 c8
y3 z3 z4 y4 z5 z6 y6 z7 y7 z8 z9 y9 z10
NQGYYDVKPR+XL_S
A_b_ion
A_y_ion
y2+ y3+ y4+ y5+ y6+ y7+ y8+ y9+ y10+ prec
b2+ b3+ b4+ b5+ b7+ b8+ b9+
m/z
NQGYYDVKPR+XL_L
NQGYYDVKPR+XL_S
B_b_ion
B_y_ion
m/z
NQGYYDVKPR+XL_L
